# Supplementary material for: Chromothripsis during telomere crisis is independent of NHEJ, and consistent with a replicative origin
Source: Genome Res. 2019 May;29(5):737–49. doi: 10.1101/gr.240705.118 (PMC6499312; doi:10.1101/gr.240705.118)
Supplement: Supplemental Material [file supp_gr.240705.118_Supplemental_file_1.zip › contigs/annotated_contigs/DB86/contig.2.DB86_length_463_mean_cov_5.83153347732.docx]

**DB86_length_463_mean_cov_5.83153347732**

```
CACCCCTTGCCAGAGCTGGGGACCCAAAGCGCTGAGCACCGTGAGCTCTGCTCACAAGGGTGTGGCACCCTCTGGTCTGGCAAAGCCTT
 >chr5:175827482-175827712 + E=7e-127
TCCAATCCAGGTCCAGCCACACCCGCTCTGGCACCCACGCCCTCCGCATTCCTGTCTGCAGGCATCCCACTCCAGCTACCCGGCCTTTG

TCTCCTGGCGCTTCTGTCTCCCCTCCCAGCAATAGTCTCCCCTCCCTC|CTCC|GCCTCCCGGGTTCACGCCATTCTCCTGCCTCAGCC
                                                 >chr5:177412178-177412415 - E=4e-128
TCCGGAGTAGCTGGGACTACAGGCGCCCGCCACCACGCTCGGCTAATTTGTTGTATTTTCAGTAGAAACTGAGTTTCACCGTGTTAGCC

AGGATGGTCTCGATCTCCTGACCTCGTGATCCGCCCGCCTCGGCCTCCCAAAGTGTTGGGACTACAGGCGTGAGCCACCGCGCTCCGCC

GAGGAGATACTCTAGAAGCT
```
